# Supplementary material for: A live measles-vectored COVID-19 vaccine induces strong immunity and protection from SARS-CoV-2 challenge in mice and hamsters
Source: Nat Commun. 2021 Nov 1;12:6277. doi: 10.1038/s41467-021-26506-2 (PMC8560864; doi:10.1038/s41467-021-26506-2)
Supplement: Supplementary file 1 — Supplementary Information [file 41467_2021_26506_MOESM1_ESM.pdf]

Supplementary information

A live measles-vectored COVID-19 vaccine induces strong immunity and protection from SARS-CoV-2 challenge in mice and hamsters.

Phanramphoei N. Frantz[1,2], Aleksandr Barinov[1,3], Claude Ruffié[1], Chantal Combredet[1], Valérie Najburg[1], Guilherme Dias de Melo[4], Florence Larrous[4], Lauriane Kergoat[4], Samaporn Teeravechyan[2], Anan Jongkaewwattana[2], Emmanuelle Billon-Denis[5], Jean-Nicolas Tournier[5], Matthieu Prot[6], Laurine Levillayer[6], Laurine Conquet[7], Xavier Montagutelli[7], Magali Tichit[8], David Hardy[8], Priyanka Fernandes[9], Hélène Strick-Marchand[9], James Di Santo[9], Etienne Simon-Lorière[6], Hervé Bourhy[4], and Frédéric Tangy[1,†]

[1] Institut Pasteur, Université de Paris, Innovation Lab: Vaccines, Paris, France

[2] National Center for Genetic Engineering and Biotechnology (BIOTEC), National Science and Technology Development Agency, Virology and Cell Technology Laboratory, Pathumthani, Thailand

[3] Viroxis, Paris, France

[4] Institut Pasteur, Université de Paris, Lyssavirus Epidemiology and Neuropathology Unit, Paris, France

[5] Armed Forces Biomedical Research Institute (IRBA), Microbiology and infectious diseases department, Brétgny-sur-Orge, France

[6] Institut Pasteur, Université de Paris, Evolutionary Genomics of RNA viruses unit, Paris, France

[7] Institut Pasteur, Université de Paris, Laboratory of Mouse Genetics, Paris, France

[8] Institut Pasteur, Université de Paris, Experimental Neuropathology unit, Paris, France

[9] Institut Pasteur, Université de Paris, INSERM U1223, Innate Immunity unit, Paris, France

[†] Corresponding author: Frédéric Tangy
Email: frederic.tangy@pasteur.fr

SFig. 1

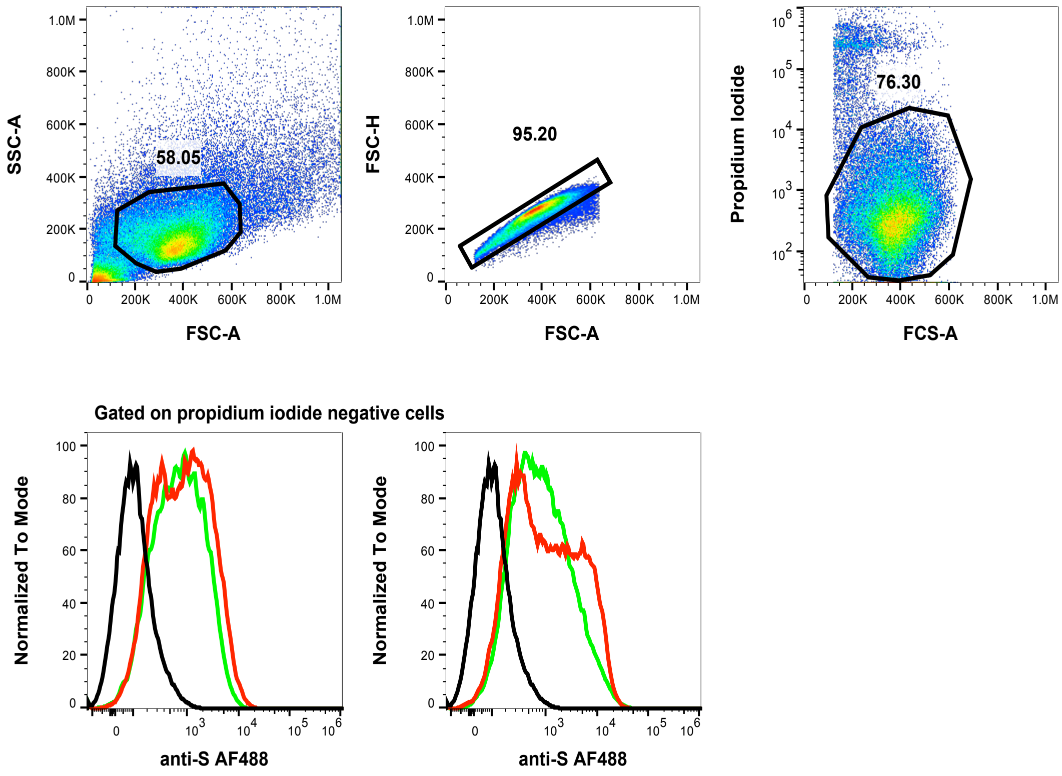

Sample	MFI
Negative control	178
SF-2P-dER	1604
SF-dER	1237

Sample	MFI
Negative control	178
S2-2P-dER	2687
S2-dER	1907

Supplementary figure 1. Expression of SARS-CoV-2 S antigens on the surface of transfected HEK293T cells. Cells transfected with pcDNA expression vectors encoding full-length S or S2 subunit antigens were stained for indirect immunofluorescence with an anti-S antibody followed by Alexa Fluor 488-conjugated goat anti-rabbit IgG. Propidium iodide was used to exclude dead cells by gating (upper dot plots). Histograms show surface expression of full-length S (left histograms) or S2 subunit proteins (right histograms). Native-conformation S antigens (green), prefusion-stabilized S (red), mock-transfected control cells (black histograms) and corresponding mean fluorescence intensities (MFI) are shown. The experiment shown was conducted using two biologically independent batches.

SFig. 2

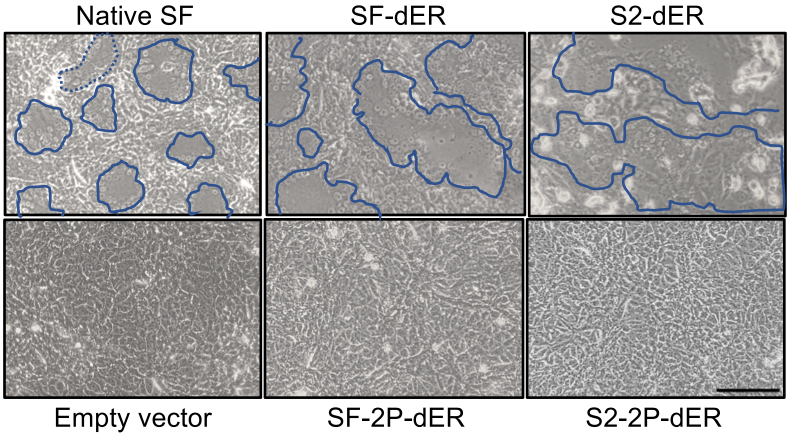

Supplementary figure 2. S protein-mediated syncytium formation in transfected Vero cells. Images of Vero cells transfected with pcDNA expression vectors encoding SARS-CoV-2 S proteins were acquired 24 hours post-transfection. Upper images show Vero cells transfected with plasmids encoding native-conformation S antigens, while lower images depict cells transfected with prefusion-stabilized S antigens and non-transfected control Vero cells. Blue lines delineate the borders of syncytia. Native SF indicates native-conformation full-length S protein with an intact CT. Microscope was used at 10x magnification (scale bar, 100 µm). The experiment shown was conducted using two biologically independent Vero cell batches.

SFig. 3

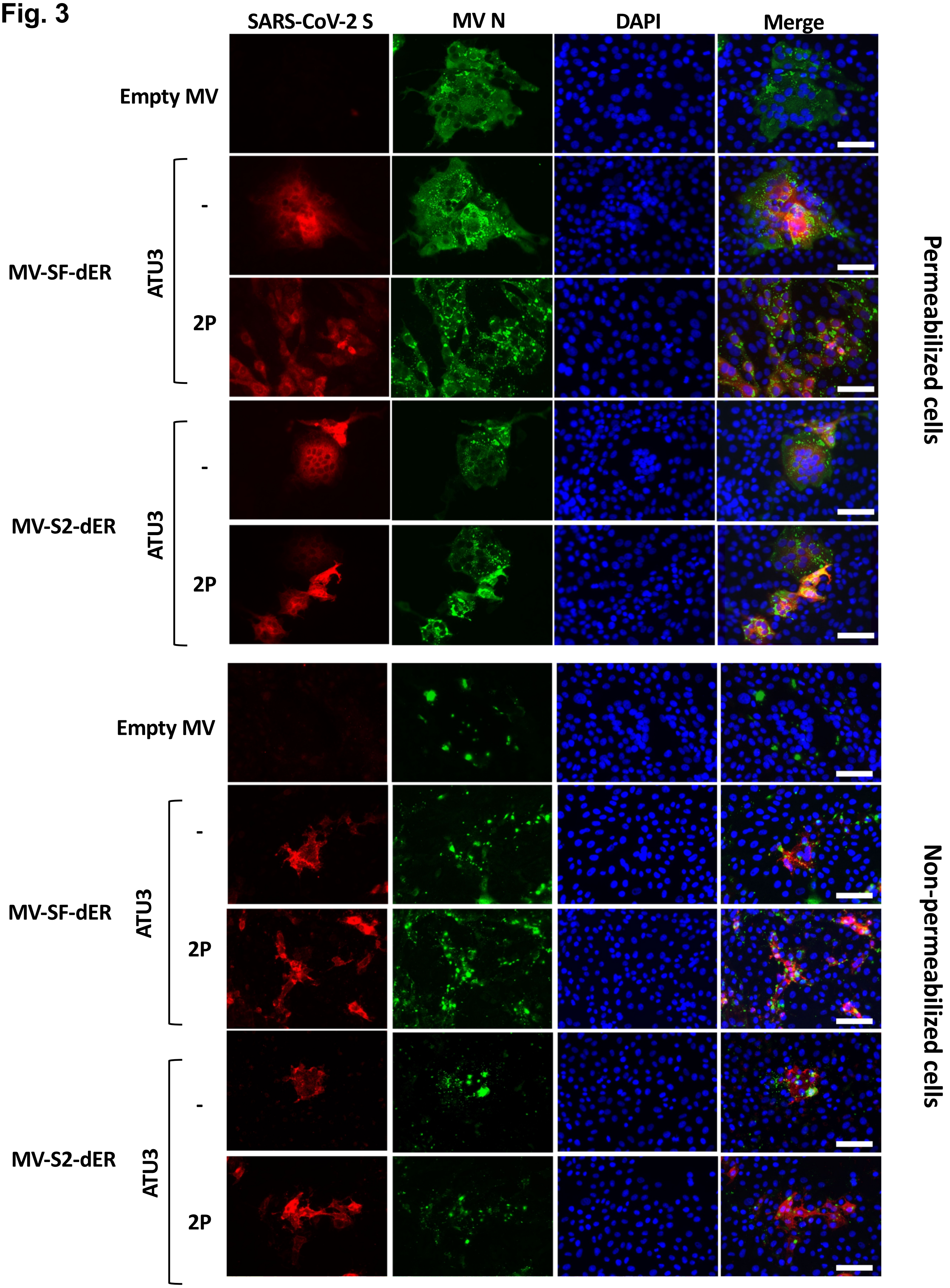

Supplementary figure 3. Immunofluorescence analysis of intracellular S protein expression in Vero cells infected with recombinant MV vaccines. Vero cells were infected with rMVs expressing SARS-CoV-2 S proteins or empty MV Schwarz. Twenty-four hours after infection, S protein was detected in saponin-permeabilized cells or non-saponin treated cells using a mouse anti-S antibody followed by Cy3-conjugated goat anti-mouse IgG (red). MV N protein was visualized using rabbit polyclonal anti-N antibody followed by Alexa Fluor 488-conjugated goat anti-rabbit IgG (green). Nuclei were stained with DAPI (blue). Images were acquired using a fluorescence microscope at 20x magnification (scale bar, 50 μm) The experiment shown was conducted using two or three biologically independent Vero cells batches.

SFig. 4

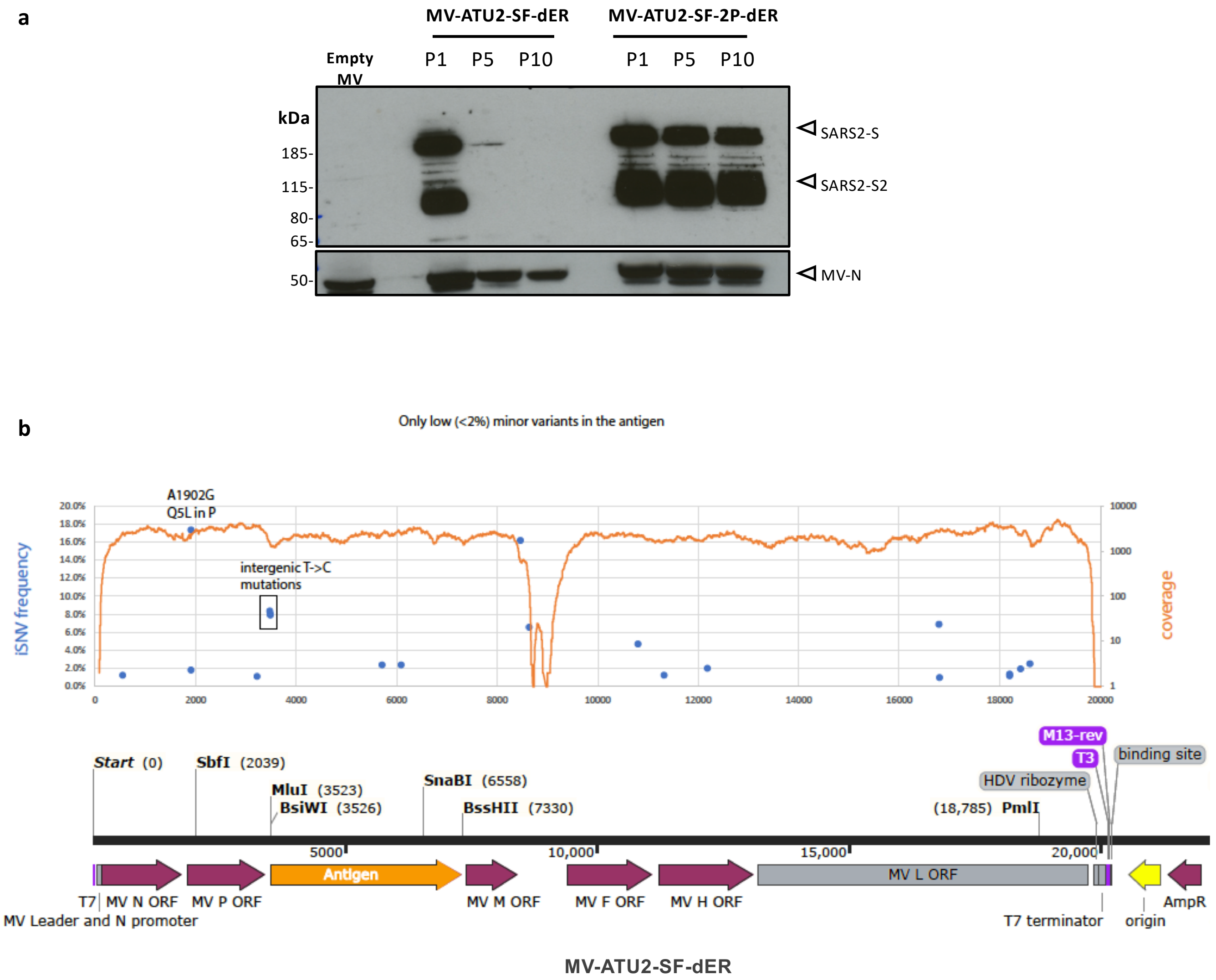

Supplementary figure 4. The genetic stability of recombinant MV vaccines from serial passages; **a**) The Western blot analysis of S protein expression in Vero cells infected with recombinant MV vaccines from serial passages. MV ATU2 vaccines expressing SF-dER or SF-2P-dER antigens were serially passaged on Vero cells from P1 up to P10 and S protein expression was determined by immunoblotting of P1, P5, and P10 cell lysates. Vero cells infected with empty MV were examined in parallel and served as negative controls. **b**) Genome coverage of MV-ATU2-SF-dER (P4) from Next-Generation Sequencing. The experiments shown were conducted using two or three biologically independent Vero cell batches.

SFig. 5

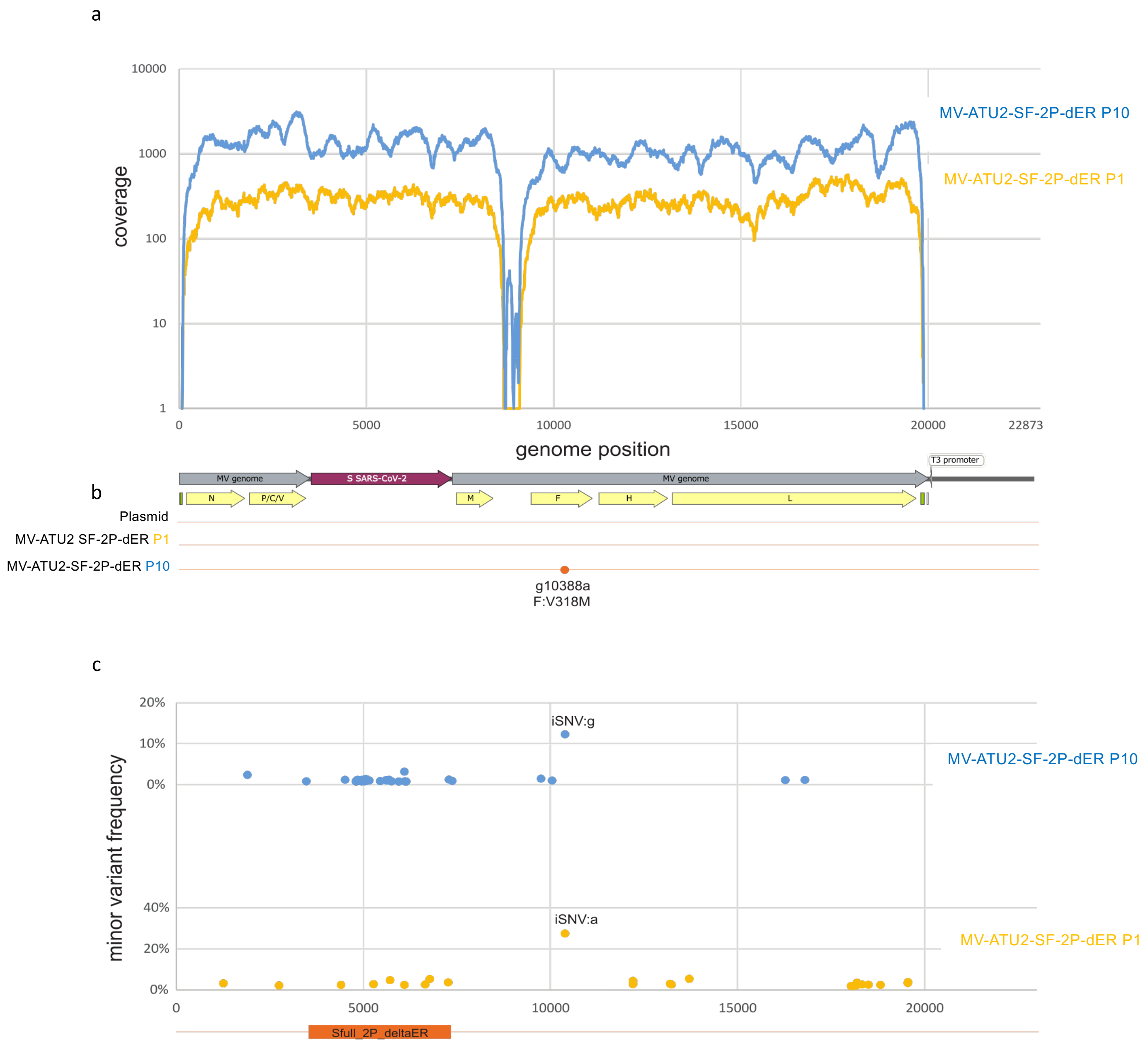

Supplementary figure 5. Genome coverage of MV-ATU2-SF-2P-dER from Next-Generation Sequencing depicted in; **a)** Schematic of genome coverage to with the genome position with yellow and blue lines represent MV-ATU2-SF-2P-dER genome sequencing of P1 and P10, respectively. **b)** Schematic of sequence comparison of plasmid to MV-ATU2-SF-2P-dER genome sequencing of P1 and P10. **c)** Percent minor variant frequency with yellow and blue dots represent MV-ATU2-SF-2P-dER genome of P1 and P10.

SFig. 6

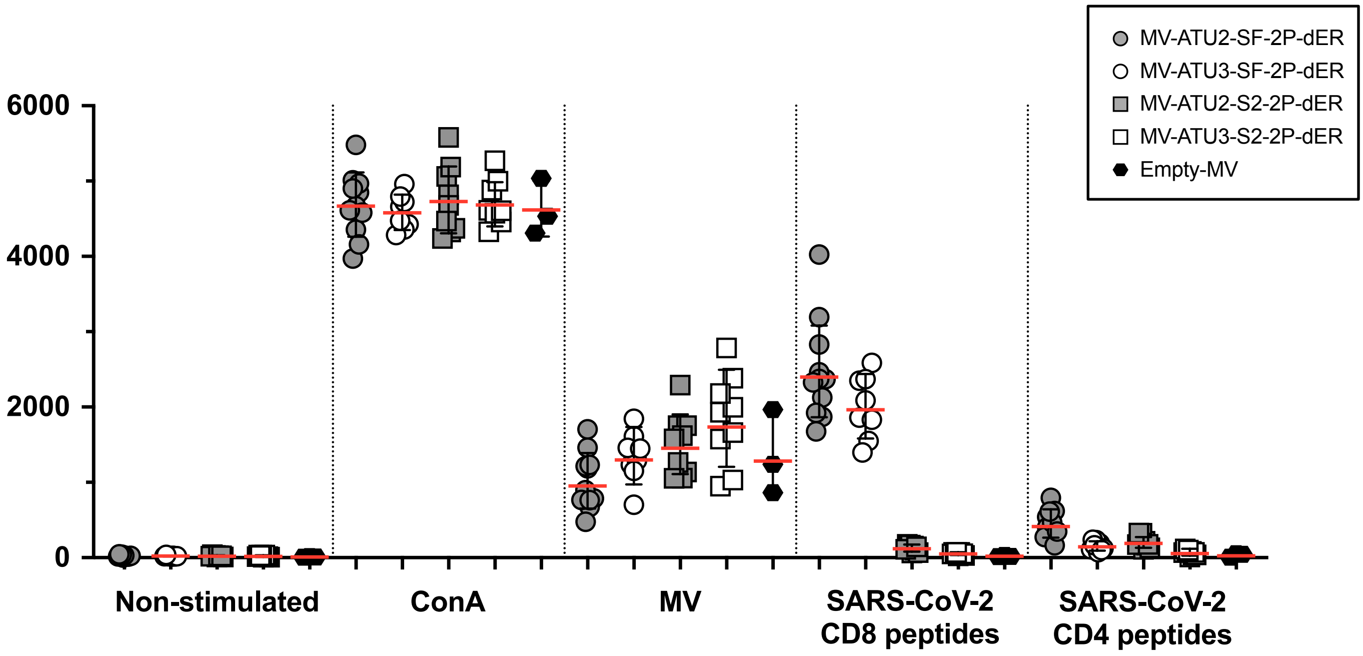

Supplementary figure 6. IFN-γ ELISPOT analysis of T cell responses elicited in IFNAR$^{-/-}$ mice immunized with rMV SARS-CoV-2 vaccine candidates. Groups of mice were immunized with MV-ATU2-SF-2P-dER, n=11; MV-ATU3-SF-2P-dER, n=8; MV-ATU2-S2-2P-dER, n=9; MV-ATU3-S2-2P-dER, n=9 and Empty MV, n=3. Splenocytes of vaccinated mice were stimulated *ex vivo* with a pool of synthetic peptides covering the predicted CD8^{+} and CD4^{+} T-cell epitopes of the SARS-CoV-2 S protein (Supplementary Table1). Splenocytes were also stimulated with an empty MV virus to detect MV vector-specific T-cell responses, concanavalin A for positive controls or were left unstimulated (negative controls). The number of IFN-γ secreting cells per $1x10^{6}$ splenocytes in individual animals are shown. Data are presented as geometric mean with lines and error bars indicating ± geometric SD.

SFig. 7

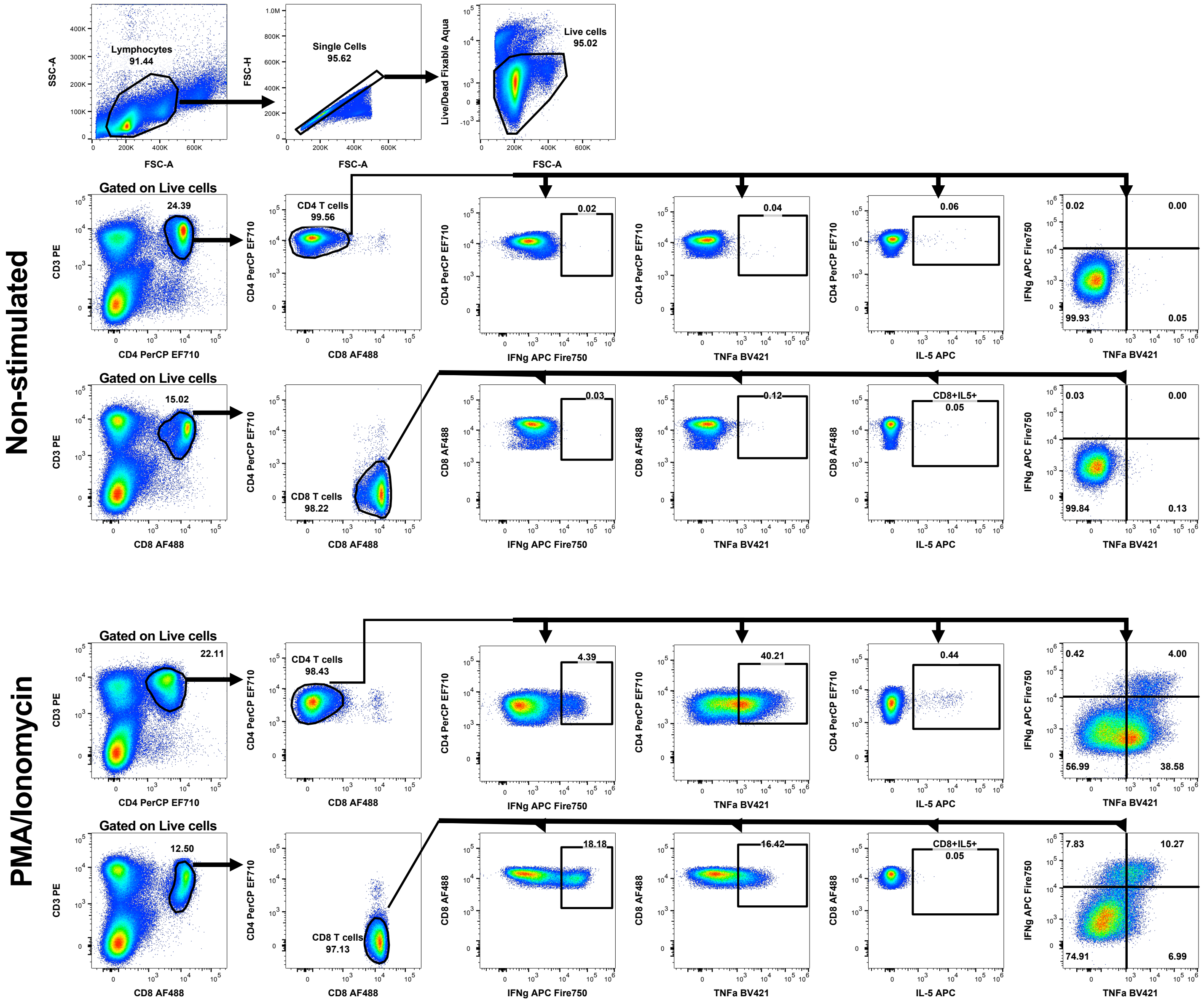

Supplementary figure 7. Gating strategy for intracellular cytokine staining. An exemplary depiction of the gating strategy to analyze IFN-γ, TNF-α and IL-5 T cell responses in non-stimulated or PMA/Ionomycin stimulated (positive controls) mouse splenocytes. Live/Dead Fixable Aqua Viability Dye was used to exclude dead cells by gating (top plots). For each stimulation group, the first two FACS plots on the left depict gating of $CD4^+$ (upper plots in each group) and $CD8^+$ T cells (lower plots). $CD4^+$ and $CD8^+$ T cells from each group were analyzed for the expression of IFN-γ, TNF-α and IL-5 (from the left, FACS plots 3, 4 and 5 respectively). These IFN-γ, TNF-α and IL-5 gates were used to analyze splenocytes stimulated with a pool of synthetic peptides covering the predicted $CD8^+$ and $CD4^+$ T-cell epitopes of the SARS-CoV-2 S protein from rMV vaccinated animals, the data of these analyses are presented in Fig.5. FACS plots on the right define quadrant IFN-γ and TNF-α gates in $CD4^+$ and CD8 T^+ cells.

SFig.8

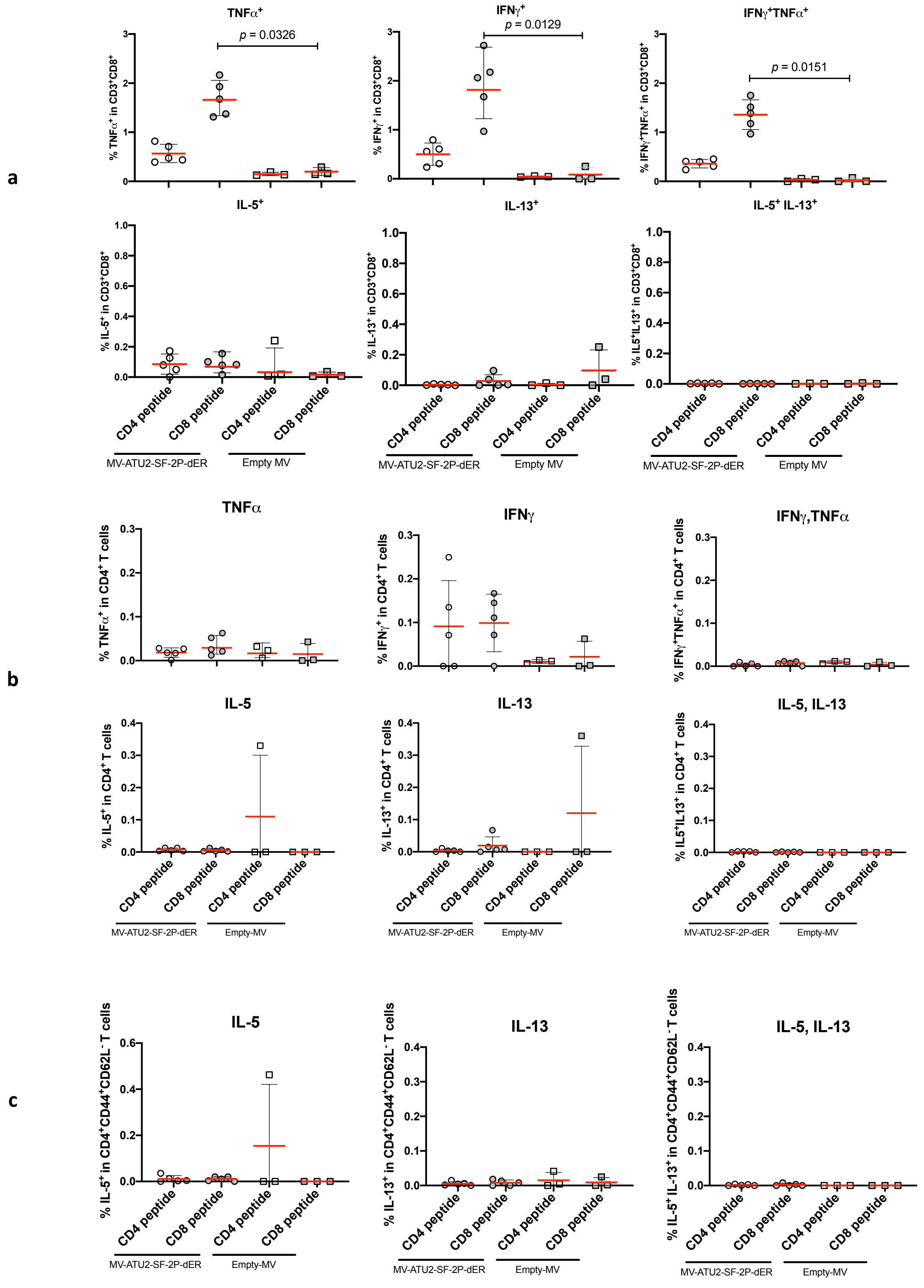

Supplementary figure 8. Cytokine expression profile of T cells assessed in IFNAR$^{-/-}$ mice (n=5 or n=3 for a control Empty MV group) immunized intraperitoneally (i.p.) with 1x10^5 TCID$_{50}$ of MV-ATU2-SF-2P-dER or Empty MV. Splenocytes were stimulated with either S-specific CD4 or CD8 peptide (S. Table1). S-specific **a)** CD8$^+$ and **b)** CD4$^+$ T-cells were stained for intracellular IFNγ, TNFα, IL-5 and IL13. **c)** S-specific CD4$^+$ memory T cells were stained for intracellular IL-5 and IL13. Data are presented as geometric mean with lines and error bars indicating ± geometric SD. Statistical difference was determined by Kruskal-Wallis one-way ANOVA test with Dunn's multiple comparison test.

SFig. 9

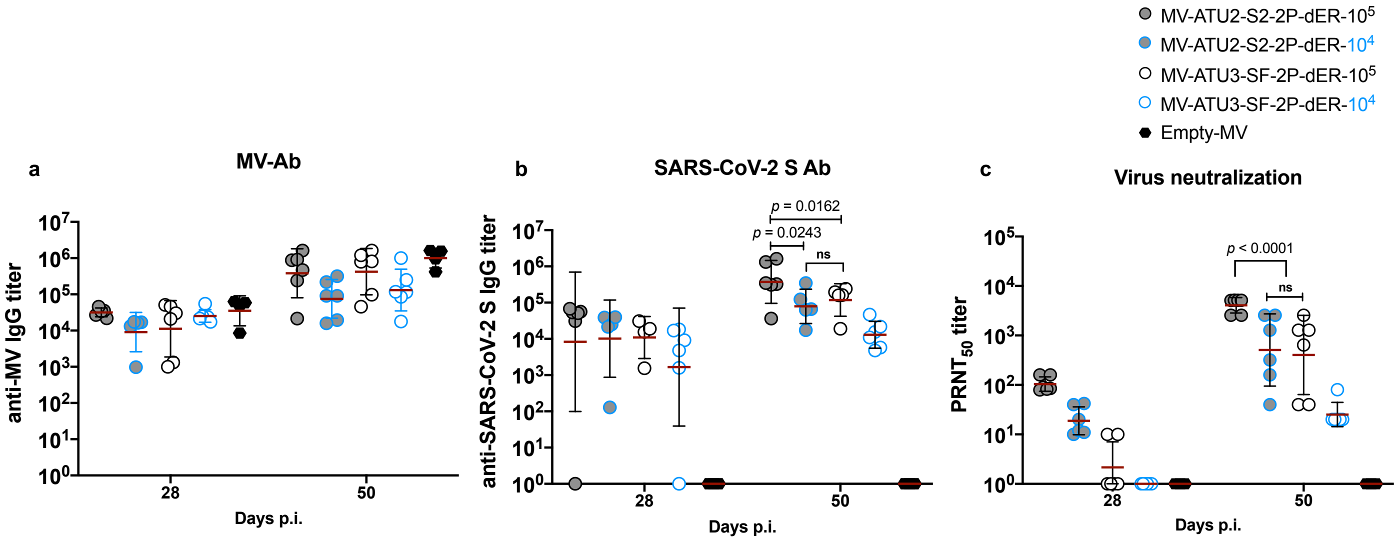

Supplementary figure 9. Dose-dependent homologous prime-boost immunization. IFNAR $^{-/-}$ mice (*n*=6 or *n*=4 for the empty MV control) were immunized intraperitoneally with the indicated rMV vaccine candidates at 1x10^5 TCID$_{50}$ or 1x10^4 TCID$_{50}$ at days 0 and 28. Sera were collected 28 and 50 days after immunization and assessed for specific antibody responses to **a)** MV antigen or **b)** SARS-CoV-2 S protein. The data show the reciprocal endpoint dilution titers with each data point represents an individual animal. **c)** Neutralizing antibody response to SARS-CoV-2 virus expressed as 50% plaque reduction neutralization test (PRNT$_{50}$) titers. Data are represented as geometric means with lines and error bars indicating ± geometric SD. Significant differences between the groups were determined by the two-tailed Mann-Whitney test.

SFig. 10

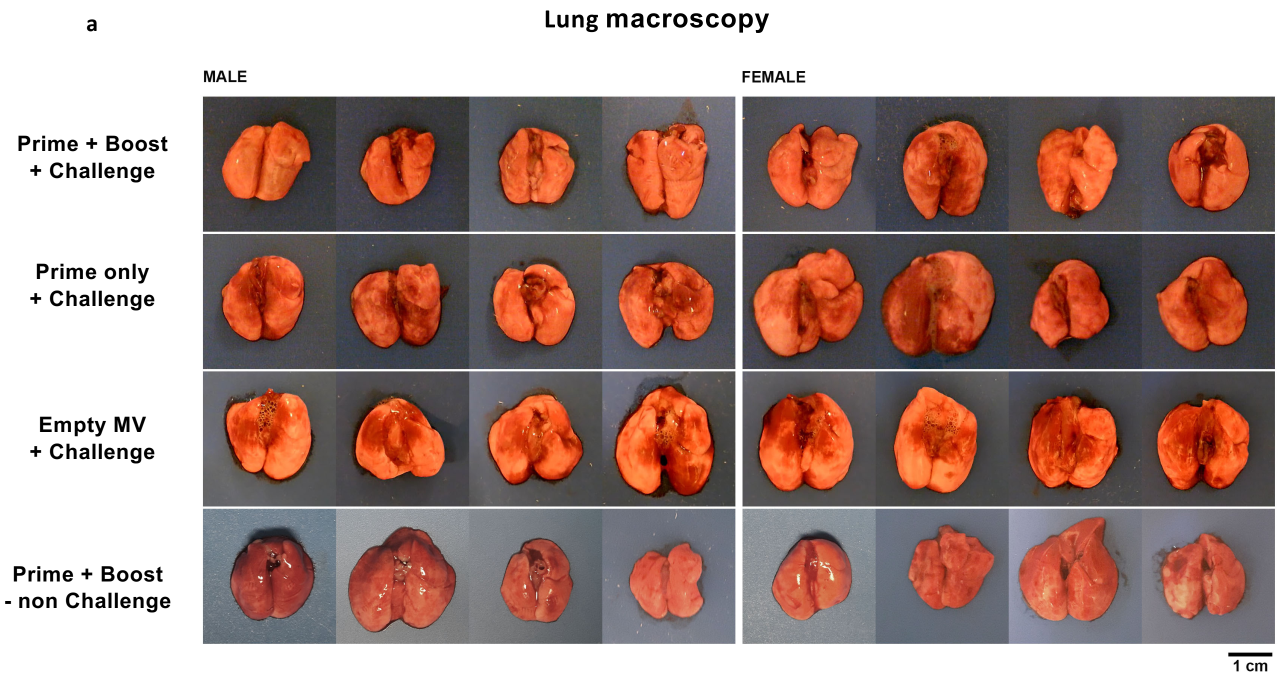

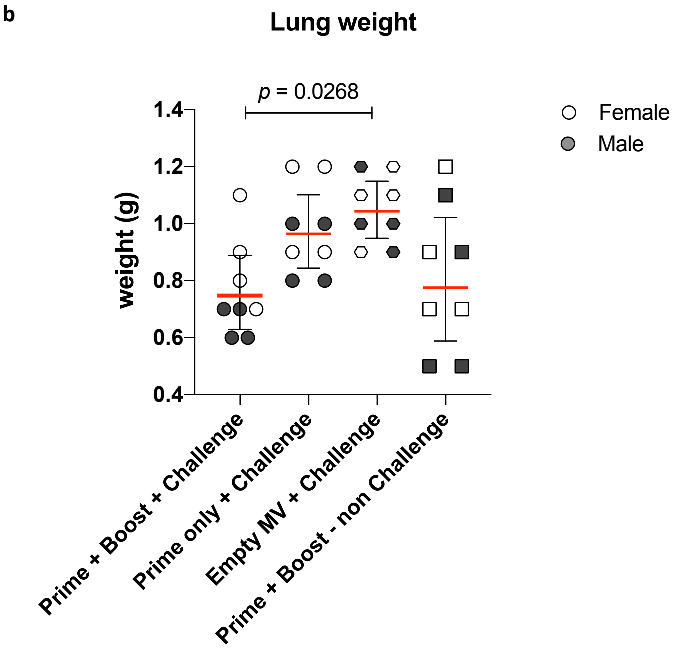

Supplementary figure 10. MV-ATU2-SF-2P-dER immunization protects SARS-CoV-2-challenged golden Syrian hamsters from lung pathology. The vaccinated animals were challenged (n=8/group). **a**) representative images of freshly-collected lungs at 4 dpi from immunized and challenged hamsters. Scale bar = 1 cm. **b)**. lung weight of the immunized and challenged animals collected at 4 dpi. Data are presented as geometric mean with lines and error bars indicating ± geometric SD. Statistical significance was determined by Kruskal-Wallis one-way ANOVA test with Dunn's multiple comparison test.

SFig. 11

```
>SF-dER DNA sequence  (3792 bp)
ATGTTCGTCTTTCTGGTATTGCTTCCTCTGGTGAGTTCCCAGTGCGTGAACCTGACGACTCGGACACAGCTGCCGCCTGCATA
CACAAACAGCTTCACACGAGGTGTTTACTATCCGGACAAGGTGTTCCGGTCCTCTGTCTTGCACAGTACTCAGGACCTTTTTC
TGCCATTCTTTAGTAACGTAACATGGTTCCACGCTATCCATGTAAGTGGAACCAATGGCACCAAGCGCTTCGACAATCCAGTC
CTGCCTTTCAACGATGGCGTCTACTTCGCCTCTACCGAGAAATCAAATATTATTAGAGGGTGGATCTTCGGGACCACCCTTGA
TAGCAAGACTCAGAGTCTTCTGATCGTGAACAATGCTACTAACGTCGTCATAAAAGTATGTGAATTTCAGTTCTGCAATGACC
CCTTCCTGGGCGTGTACTATCACAAAAATAACAAGTCCTGGATGGAATCCGAGTTTCGGGTATACAGCAGCGCTAATAACTGT
ACCTTCGAATATGTGAGCCAGCCATTCCTGATGGACCTGGAGGGGAAGCAGGGCAACTTCAAAAACCTGAGGGAATTCGTGTT
CAAGAACATTGACGGATACTTCAAAATCTACAGCAAGCACACTCCAATTAATCTTGTTAGGGACCTTCCTCAGGGGTTCTCCG
CGCTGGAACCCTTGGTGGATCTGCCAATTGGTATTAACATCACGAGGTTTCAAACCCTGCTCGCCCTCCACCGGTCATACCTG
ACCCCAGGTGATAGTAGTAGTGGCTGGACAGCAGGGGCCGCAGCATACTACGTCGGCTACCTCCAGCCCCGCACATTCCTGCT
GAAGTATAATGAAAACGGTACTATTACAGATGCTGTGGACTGCGCACTTGATCCATTGTCCGAGACTAAATGCACCTTGAAGT
CTTTTACCGTTGAGAAGGGAATCTATCAGACCAGTAATTTCAGAGTGCAACCCACCGAGAGCATCGTGCGATTTCCTAATATT
ACAAACCTGTGCCCTTTTGGCGAGGTGTTTAACGCCACTCGCTTCGCGAGCGTGTATGCATGGAACCGGAAAAGGATTAGCAA
CTGCGTGGCTGATTATTCAGTCCTTTATAATAGTGCCTCTTTCAGTACGTTCAAATGCTATGGAGTGTCCCCAACTAAGCTTA
ACGATCTGTGTTTTACCAACGTGTATGCCGACTCTTTCGTCATCAGGGGCGACGAGGTTAGACAAATTGCGCCCGGACAGACA
GGCAAAATTGCTGACTACAATTATAAACTGCCAGATGACTTTACAGGATGCGTGATTGCCTGGAACAGCAACAACCTTGATTC
TAAGGTGGGCGGAAACTACAACTATCTGTACAGGCTGTTCCGCAAATCAAACCTGAAGCCATTCGAAAGGGACATTTCAACTG
AGATCTACCAGGCCGGATCTACACCTTGCAACGGGGTGGAAGGATTCAACTGCTACTTCCCGCTCCAGTCATATGGCTTTCAA
CCTACCAATGGAGTGGGCTACCAACCTTATCGAGTAGTTGTTCTCTCTTTCGAGTTGTTGCATGCCCCAGCCACTGTCTGCGG
ACCAAAGAAATCCACCAATCTGGTGAAAAACAAGTGCGTCAACTTCAACTTCAATGGACTCACCGGTACAGGGGTTCTTACCG
AGTCAAACAAGAAATTCCTTCCGTTTCAGCAGTTTGGACGCGATATCGCCGACACTACGGATGCAGTACGCGATCCTCAGACA
CTTGAGATCCTGGACATCACTCCATGCTCTTTTGGAGGGGTGTCTGTCATCACTCCCGGGACGAATACCAGTAATCAGGTCGC
CGTGCTCTATCAAGATGTCAACTGCACAGAGGTGCCAGTCGCCATTCACGCCGATCAGCTTACCCCTACTTGGCGAGTATATA
GCACAGGGTCCAACGTGTTTCAGACAAGGGCGGGTTGTCTGATCGGCGCTGAGCACGTCAATAATAGCTACGAATGTGACATC
CCTATTGGAGCCGGGATTTGCGCTAGCTATCAGACTCAGACTAACTCACCCCGGCGAGCTAGGTCTGTAGCAAGTCAGAGTAT
CATAGCCTATACTATGAGCCTGGGGGCAGAAAATTCTGTCGCTTATAGTAATAACAGTATTGCAATTCCTACTAACTTCACTA
TTAGCGTGACTACAGAAATTCTGCCAGTGAGCATGACCAAGACCTCCGTCGACTGTACCATGTATATCTGCGGAGACAGCACA
GAATGCAGCAACCTGCTTCTGCAGTACGGTAGCTTTTGTACCCAGCTGAACAGGGCACTGACCGGCATCGCTGTGGAACAGGA
CAAAAATACGCAGGAGGTGTTCGCTCAGGTCAAGCAGATTTATAAGACACCCCCAATAAAAGACTTCGGCGGCTTTAACTTCT
CCCAGATCCTGCCCGATCCGTCAAAACCTTCCAAGAGAAGTTTTATCGAGGATCTGCTGTTTAACAAAGTAACATTGGCTGAT
GCTGGCTTTATTAAACAGTACGGCGATTGTCTGGGAGATATCGCCGCTAGAGATCTGATCTGCGCTCAAAAATTTAATGGCCT
CACTGTATTGCCCCCCCTGCTGACGGATGAAATGATTGCCCAATACACATCAGCCCTGCTGGCTGGGACAATCACTTCCGGCT
GGACGTTCGGTGCTGGAGCTGCTCTTCAAATTCCATTTGCCATGCAAATGGCTTATAGATTCAACGGAATTGGCGTGACCCAA
AACGTGCTGTACGAAAATCAAAAGCTGATTGCTAATCAGTTTAACTCCGCTATCGGTAAGATACAGGACAGCCTGTCTAGTAC
TGCCAGCGCCCTCGGAAAGCTCCAGGATGTGGTGAACCAGAATGCGCAGGCGCTGAACACACTCGTCAAACAGCTTAGCAGCA
ATTTCGGCGCAATTAGCAGTGTACTGAACGACATCTTGTCTCGGCTCGACAAGGTCGAGGCCGAAGTGCAGATTGATCGCCTG
ATCACTGGCCGGCTCCAGTCTCTGCAGACCTACGTAACCCAGCAACTCATACGCGCTGCCGAAATTCGGGCTTCAGCTAACCT
GGCCGCAACAAAGATGTCTGAATGTGTTCTTGGGCAGTCCAAGAGGGTGGACTTCTGCGGGAAAGGATATCACCTGATGTCCT
TCCCTCAGAGCGCCCCTCATGGAGTGGTGTTTCTTCATGTTACCTATGTTCCGGCCCAGGAAAAGAATTTTACCACTGCCCCA
GCCATTTGCCATGATGGTAAAGCACACTTCCCAAGAGAGGGCGTGTTTGTTAGTAACGGCACCCACTGGTTCGTTACCCAGCG
CAATTTTTACGAACCTCAAATCATTACCACTGACAATACATTTGTATCAGGCAATTGCGACGTGGTGATCGGAATCGTAAATA
ATACAGTCTACGATCCCCTGCAGCCAGAGCTTGACAGCTTTAAAGAGGAACTGGACAAATACTTCAAAAATCATACAAGCCCC
GACGTCGACCTGGGAGATATTTCTGGCATCAATGCCTCCGTCGTGAATATCCAAAAAGAGATCGACCGCCTTAATGAGGTTGC
CAAAAACCTCAACGAGTCCCTGATTGATCTGCAGGAGCTGGGGAAGTACGAGCAGTATATCAAATGGCCCTGGTACATCTGGC
TGGGCTTCATTGCCGGGCTCATAGCCATCGTGATGGTGACGATCATGCTGTGTTGCATGACCTCTTGCTGTTCTTGTCTGAAG
GGGTGCTGCTCTTGTGGGAGTTGTTGTAAATTCGATGAGGATGATTCCGAATAATAG
```

Supplementary figure 11. DNA sequence of SARS-CoV-2 SF-dER used as the template to construct SARS-CoV-2 vaccine candidates.

S.Table 1

Supplementary Table1. Peptide pools corresponding to the S1 and S2 subunits used to stimulate S-specific CD4$^+$ or CD8$^+$ T cells.

CD4 peptides	Subunit	Amino acid position
QDLFLPFFSNVTWFH	S1	52-66
STEIYQAGSTPCNGV	S1	469-483
VLSFELLHAPATVCG	S1	512-526
ENSVAYSNNSIAIPT	S2	702-716
ITSGWTFGAGAALQI	S2	882-896
QMAYRFNGIGVTQNV	S2	901-915
GKIQDSLSSTASALG	S2	932-946
IRAAEIRASANLAAT	S2	1013-1027
GYHLMSFPQSAPHGV	S2	1046-1060
PAQEKNFTTAPAICH	S2	1069-1083

CD8 peptides	Subunit	Amino acid position
FVFLVLLPL	S1	2-10
VNLTTRTQL	S1	16-24
LFLPFFSNV	S1	54-62
SNVTWFHAI	S1	60-68
VTWFHAIHV	S1	62-70
RGWIFGTTL	S1	102-110
FQFCNDPFL	S1	133-141
YSSANNCTF	S1	160-168
VSQPFLMDL	S1	171-179
KIYSKHTPI	S1	202-210
INITRFQTL	S1	233-241
AAAYYVGYL	S1	262-270
VRFPNITNL	S1	327-335
FNATRFASV	S1	342-350
GNYNYLYRL	S1	447-455
VGYQPYRVV	S1	503-511
VVVLSFELL	S1	510-518
VNFNFNGLT	S1	539-547
YQDVNCTEV	S1	612-620
SIIAYTMSL	S2	691-699
VAYSNNSIA	S2	705-713
FGGFNFSQI	S2	797-805
AALQIPFAM	S2	892-900
VVNQNAQAL	S2	951-959
VVFLHVTYV	S2	1060-1068
ISGINASVV	S2	1169-1177
IWLGFIAGL	S2	1216-1224
IAIVMVTIM	S2	1225-1233

S.Table 2

Supplementary Table 2. Primers used for construction and sequencing of SF-dER, S2-dER and their 2P mutation counterparts along with the primers used for mouse-adapted SARS-CoV-2 vRNA detection.

Primer name	Sequence
**Construction primers**	
BsiWI-Signal	TAACGTACGGCCACCATGTTCGTCTTTCTGGTATTG
BssHII-SF	TAAGCGCGCCTATTATTCGGAATCATCCTCATCGA
BsmBI-signal	TAACGTCTCCGCACTGGGAACTCACCAGAGGAAG
BsmBI-S2	TAACGTCTCAGTGCGTAGCAAGTCAGAGTATCATAG
BssHII-S2	TAAGCGCGCTTATTCGGAATCATCCTCATCGAATTTAC
BsmBI-2P-fwd	AATCGTCTCACACCAGAGGCCGAAGTGCAGATTGATCGCCTG
BsmBI-2P-rev	ATACGTCTCAGGTGGGTCGAGCCGAGACAAGATGTCGTTC
BsmBI-deltaF-fwd	AATCGTCTCTCCTCTGTAGCAAGTCAGAGTATCATAG
BsmBI-deltaF-rev	ATACGTCTCTGAGGGTGAGTTAGTCTGAGTCTGATAG
**Sequencing primers**	
Signal-fwd1	TCTGGTATTGCTTCCTCTGGTG
SF-fwd2	TGCGCACTTGATCCATTGTC
S2-fwd3	GTAAAGCACACTTCCCAAGAG
SF-fwd4	GATCCTGGACATCACTCCATGC
SF-rev1	TTCCACTTACATGGATAGCGTGG
S2-rev1	TACTGTTATTACTATAAGCGACAG
S2-rev3	GATCTCTAGCGGCGATATCTC
3433	GACCTTGGGAGGCAATCACT
oligo8a	GGAATCGCTGTCCTCAACAA
9119	AGATAGGGCTGCTAGTGAACCAAT
9218	TGGACCCTACGTTTTTCTTAATTCT
**qRT-PCR primers**	
**MACo3**	
nCoV_IP4-14059Fw	GGTAACTGGTATGATTTCG
nCoV_IP4-14146Rv	CTGGTCAAGGTTAATATAGG
nCoV_IP4-14084Probe(+)	TCATACAAACCACGCCAGG [5']Hex [3']BHQ-1 19
**SARS-CoV-2**	
nCoV_IP2-12669Fw	ATGAGCTTAGTCCTGTTG
nCoV_IP2-12759Rv	CTCCCTTTGTTGTGTTGT
nCoV_IP2 probe	AGATGTCTTGTGCTGCCGGTA [5']FAM [3']TAMRA
