# Supplementary material for: A live measles-vectored COVID-19 vaccine induces strong immunity and protection from SARS-CoV-2 challenge in mice and hamsters
Source: Nat Commun. 2021 Nov 1;12:6277. doi: 10.1038/s41467-021-26506-2 (PMC8560864; doi:10.1038/s41467-021-26506-2)
Supplement: Supplementary file 3 — Reporting Summary [file 41467_2021_26506_MOESM3_ESM.pdf]

nature research

Corresponding author(s): Frederic Tangy

Last updated by author(s): Sep 29, 2021

Reporting Summary

Nature Research wishes to improve the reproducibility of the work that we publish. This form provides structure for consistency and transparency in reporting. For further information on Nature Research policies, see our Editorial Policies and the Editorial Policy Checklist.

Statistics

For all statistical analyses, confirm that the following items are present in the figure legend, table legend, main text, or Methods section.

n/a	Confirmed	
☐	☒	The exact sample size (n) for each experimental group/condition, given as a discrete number and unit of measurement
☐	☒	A statement on whether measurements were taken from distinct samples or whether the same sample was measured repeatedly
☐	☒	The statistical test(s) used AND whether they are one- or two-sided<br>*Only common tests should be described solely by name; describe more complex techniques in the Methods section.*
☒	☐	A description of all covariates tested
☒	☐	A description of any assumptions or corrections, such as tests of normality and adjustment for multiple comparisons
☐	☒	A full description of the statistical parameters including central tendency (e.g. means) or other basic estimates (e.g. regression coefficient) AND variation (e.g. standard deviation) or associated estimates of uncertainty (e.g. confidence intervals)
☐	☒	For null hypothesis testing, the test statistic (e.g. F, t, r) with confidence intervals, effect sizes, degrees of freedom and P value noted<br>*Give P values as exact values whenever suitable.*
☒	☐	For Bayesian analysis, information on the choice of priors and Markov chain Monte Carlo settings
☒	☐	For hierarchical and complex designs, identification of the appropriate level for tests and full reporting of outcomes
☒	☐	Estimates of effect sizes (e.g. Cohen's d, Pearson's r), indicating how they were calculated

Our web collection on statistics for biologists contains articles on many of the points above.

Software and code

Policy information about availability of computer code

Data collection	Flow cytometry data were collected and analyzed using FlowJo v10.7 software (FlowJo LLC)
Data analysis	All data were analyzed using the software Prism 9 version 9.1.2

For manuscripts utilizing custom algorithms or software that are central to the research but not yet described in published literature, software must be made available to editors and reviewers. We strongly encourage code deposition in a community repository (e.g. GitHub). See the Nature Research guidelines for submitting code & software for further information.

Data

Policy information about availability of data

All manuscripts must include a data availability statement. This statement should provide the following information, where applicable:

- Accession codes, unique identifiers, or web links for publicly available datasets
- A list of figures that have associated raw data
- A description of any restrictions on data availability

Accession codes: The SARS-CoV-2 spike (S) gene, NC_045512 8
https://www.ncbi.nlm.nih.gov/nuccore/NC_045512,

The source data file with all RAW data for every figure was uploaded to Figshare;
https://figshare.com/s/c403a2a9c66d0aa1f799 (DOI: 10.6084/m9.figshare.14959518)

Field-specific reporting

Please select the one below that is the best fit for your research. If you are not sure, read the appropriate sections before making your selection.

- [x] Life sciences
- [ ] Behavioural & social sciences
- [ ] Ecological, evolutionary & environmental sciences

For a reference copy of the document with all sections, see nature.com/documents/nr-reporting-summary-flat.pdf

Life sciences study design

All studies must disclose on these points even when the disclosure is negative.

Sample size	No sample size calculations were used to power each study. Sample size for the mouse studies were determined based upon previous experience with similar studies where 5-10 animals per group represented a sufficient sample size to detect statistical differences between experimental groups [REFs to PMID: 17869338 and PMID: 23742993]. Sample size was also limited by space available in an animal ABSL-3 containment facility.
Data exclusions	No data was excluded
Replication	Animal experiments were repeated 3 times in different settings. Biological assays (ELISA, PRNT, ELISPOT, ICS) were performed in triplicate for each point and repeated 2 or 3 times. Cell infection or transfection for western blot, IF, growth curve was conducted conducted using at least two or three biologically independent Vero cell batches with reproducible data.
Randomization	Animals were allocated to the different groups by similarity of age and sex.
Blinding	The technicians performing the assays were blinded to group association and groups of samples were analyzed without knowledge of their origin.

Reporting for specific materials, systems and methods

We require information from authors about some types of materials, experimental systems and methods used in many studies. Here, indicate whether each material, system or method listed is relevant to your study. If you are not sure if a list item applies to your research, read the appropriate section before selecting a response.

Materials & experimental systems

n/a	Involved in the study	
[ ]	[x]	Antibodies
[ ]	[x]	Eukaryotic cell lines
[x]	[ ]	Palaeontology and archaeology
[ ]	[x]	Animals and other organisms
[ ]	[x]	Human research participants
[x]	[ ]	Clinical data
[x]	[ ]	Dual use research of concern

Methods

n/a	Involved in the study	
[x]	[ ]	ChIP-seq
[ ]	[x]	Flow cytometry
[x]	[ ]	MRI-based neuroimaging

Antibodies

Antibodies used	mouse monoclonal anti-SARS-CoV S antibody (Clone 1A9, ab273433, Abcam, lot GR3447576-1);<br>Alexa Fluor 488-conjugated goat anti-rabbit IgG (A-11008, ThermoFisher, lot 571716);<br>Cy3-conjugated goat anti-rabbit (A10520,115.166.072, Jackson ImmunoResearch, lot 114386);<br>rabbit polyclonal anti-S antibody targeting S2(ABIN199984), lot 181905;<br>HRP-conjugated goat anti-mouse IgG (H+L) antibody(Jackson ImmunoResearch, 115-035-146) lot 154319;<br>HRP-conjugated isotype-specific (IgG1 or IgG2a) goat anti-mouse antibodies (AB97240 lot GR3320187-9 and AB97245, lot GR3362355-1, Abcam);<br>anti-mouse IFN- γ (551216, BD Biosciences, lot 0314426);<br>anti-mouse IFN- γ antibody (554410, BD Biosciences, lot 3107795);<br>anti-CD3e PE (clone 145-2C11, 12-0031-83, eBioscience, lot 2126756);<br>anti-CD4 PerCP-eFluor710 (clone RM4-5, 46-0042-82, Invitrogen, lot 2212383);<br>anti-CD8 Alexa Fluor488 (clone 53-6.7, 53-0081-82, Invitrogen, lot 2142313);<br>anti-IFN- γ APC/Fire750, (clone XMG1.2, 505860, BioLegend, lot 13289871);<br>anti-TNF- α BV421 (cloneMP6-XT22, 563387, BD Horizon, lot 0016054);<br>anti- IL-5 APC (clone TRFK5, 505860, BD Biosciences);<br>anti-IL-13 eFluor 660 (clone eBio13A,50-7133-82, eBioscience);<br>anti-CD62L APC-Cy7 (clone MEL-14, 104427, BioLegend);<br>anti-CD44 BV786 (clone IM7, 563736, BDPharmingen).

Validation	mouse monoclonal anti-SARS-CoV S antibody (Abcam, ab273433, Clone 1A9, lot:GR3347576-1);<br>WB: Infected Caco-2 cells; SARS-CoV-2 transfected HEK-293T cell lysate; SARS-Cov1 spike protein transfected Expi cell lysate, SARS-Cov1 3xFlag spike protein transfected Expi cell lysate, SARS-Cov2 spike protein transfected Expi cell lysate and SARS-Cov2 3xFlag spike protein transfected Expi cell lysate. ICC: SARS-CoV-2 transfected BHK-21, Vero E6, COS-7 cells. IP: SARS-CoV-2 transfected HEK-293T cells. Flow Cyt: Infected HEK-293T cells.<br><br>rabbit polyclonal anti-S antibody targeting S2(ABIN199984);<br>Full length Spike protein transfected into UM92 cells was used as a positive control and an approximate 139 kD was observed<br><br>anti-mouse IFN- γ (551216, BD Biosciences) Reference PMID:1387110<br>anti-mouse IFN- γ antibody (554410, BD Biosciences) reference PMID: 1387110<br>anti-CD3e PE (clone 145-2C11, 12-0031-83, eBioscience)<br>The 145-2C11 antibody has been tested by flow cytometric analysis of mouse thymocytes and splenocytes.<br>anti-CD4 PerCP-eFluor710 (clone RM4-5, 46-0042-82, Invitrogen)<br>RM4-5 antibody has been tested by flow cytometric analysis of mouse spleen cells.<br><br>anti-CD8 Alexa Fluor488 (clone 53-6.7, 53-0081-82, Invitrogen)<br>53-6.7 antibody has been tested by flow cytometric analysis of mouse thymocyte or splenocyte<br><br>anti-IFN- γ APC/Fire750, (clone XMG1.2, 505860, BioLegend)<br>Each lot of this antibody is quality control tested by intracellular immunofluorescent staining with flow cytometric analysis. For flow cytometric staining, the suggested use of this reagent is ≤ 0.25 µg per million cells in 100 µl volume. It is recommended that the reagent be titrated for optimal performance for each application.<br><br>anti-TNF- α BV421 (cloneMP6-XT22, 563387, BD Horizon) Reference PMID: 18432811<br>anti- IL-5 APC (clone TRFK5, 505860, BD Biosciences) Reference PMID: 1387110<br>anti-IL-13 eFluor 660 (clone eBio13A,50-7133-82, eBioscience) Reference PMID: 26840450<br>anti-CD62L APC-Cy7 (clone MEL-14, 104427, BioLegend) Reference PMID: 28650992<br>anti-CD44 BV786 (clone IM7, 563736, BDPharmingen) Reference PMID:30054204

Eukaryotic cell lines

Policy information about cell lines

Cell line source(s)	Human embryonic kidney cells (HEK) 293T cells (ATCC CRL-3216); HEK293T7-NP helper cells stably expressing MV-N and MV-P genes (Tangy F, EU patent, 2006 Institut Pasteur); African green monkey kidney cells (Vero) and Vero C1008 clone E6 (ATCC CRL-1586)
Authentication	Cell lines from ATCC were not indepentdently authenticated. HEK293T7-NP helper cells stably expressing MV-N and MV-P genes were authenticated by westernblotting for expression of N and P, and the activity of T7 RNA polymerase was determined by transfection with a reporter plasmid containing the firefly luciferase coding region under the control of a T7promoter.
Mycoplasma contamination	All cell lines were tested negative for mycoplasma.
Commonly misidentified lines<br>(See ICLAC register)	No misidentified cell lines used in this study

Animals and other organisms

Policy information about studies involving animals; ARRIVE guidelines recommended for reporting animal research

Laboratory animals	129sv IFNAR-/- mice, female, 6-8 weeks old<br>Golden Syrian hamsters, aged 5-6 weeks divided into 4 groups(4 females and 4 males/group)
Wild animals	The study did not involve wild animals
Field-collected samples	The study did not involve sample collection from the field
Ethics oversight	All animal experiments were performed according to French legislation in compliance with the European Communities Council Directives (2010/63/UE, French Law 2013–118, February 6, 2013) and according to the regulations of Institut Pasteur Animal Care Committees. The Animal Experimentation Ethics Committee (CETEA 89) of the Institut Pasteur approved this study (200023) before experiments were initiated. The animals were manipulated in class III safety cabinets in the Institut Pasteur animal facilities accredited by the French Ministry of Agriculture for performing experiments on live rodents.

Note that full information on the approval of the study protocol must also be provided in the manuscript.

Human research participants

Policy information about studies involving human research participants

Population characteristics	Healthcare workers age variable from 25-60 years old exposing to first wave of SARS-CoV2 outbreak in France with the ratio 50/50 of male and female.
Recruitment	The main initial goal of this cohort was to follow the length of the serological and cellular immune response among health care workers after one of the first important cluster in an hospital setting during the first wave of COVID-19 in France.
Ethics oversight	Human sera from convalescent patients were supplied by Armed Forces Biomedical Research Institute (IRBA) from the cohort study IMMUNO-COVID-PERCY approved by a research ethics committee.

Note that full information on the approval of the study protocol must also be provided in the manuscript.

Flow Cytometry

Plots

Confirm that:

- [x] The axis labels state the marker and fluorochrome used (e.g. CD4-FITC).
- [x] The axis scales are clearly visible. Include numbers along axes only for bottom left plot of group (a 'group' is an analysis of identical markers).
- [x] All plots are contour plots with outliers or pseudocolor plots.
- [x] A numerical value for number of cells or percentage (with statistics) is provided.

Methodology

Sample preparation	Mice splenocytes were prepared after homogenization of spleens and cultivated with stimulant agents in culture medium.
Instrument	Attune NxT flow cytometer (Invitrogen)
Software	FlowJo v10.7 software (FlowJo LLC)
Cell population abundance	We did not do any sorting
Gating strategy	FCS/SSC gate was applied to identify lymphocytes, FSC-A and FSC-H to gate single cells populations, and Live/Dead Fixable Aqua vs FSC-A gate was used to identify live cells. Live cells were gated using CD3/CD4 and CD4/CD8 gates to identify CD8negative CD3+CD4+ T cells. In parallel, live cells were gated using CD3/CD8 and CD4/CD8 gates to identify CD4negative CD3+CD8+ T cells. T cell populations were defined based on clear surface marker staining pattern allowing to distinguish positive and negative populations. IFNg, TNFa and IL-5 cytokine gates were used for each of the CD4 and CD8 T-cell populations to identify cytokine secreting cells. Cytokine gates were set according to the staining pattern that allows to distinguish positive and negative populations in PMA/Ionomycin and non-stimulated cells respectively, as defined in SFig7.

- [x] Tick this box to confirm that a figure exemplifying the gating strategy is provided in the Supplementary Information.
